# Resting-State fMRI to Identify the Brain Correlates of Treatment Response to Medications in Children and Adolescents With Attention-Deficit/Hyperactivity Disorder: Lessons From the CUNMET Study

**DOI:** 10.3389/fpsyt.2021.759696

**Published:** 2021-11-16

**Authors:** Victor Pereira-Sanchez, Alexandre R. Franco, Pilar de Castro-Manglano, Maria A. Fernandez-Seara, Maria Vallejo-Valdivielso, Azucena Díez-Suárez, Miguel Fernandez-Martinez, M. Reyes Garcia de Eulate, Michael Milham, Cesar A. Soutullo, Francisco X. Castellanos

**Affiliations:** ^1^Department of Child and Adolescent Psychiatry, New York University (NYU) Grossman School of Medicine, New York, NY, United States; ^2^Departamento de Psiquiatría y Psicología Clínica, Clínica Universidad de Navarra, Pamplona, Spain; ^3^Center for the Developing Brain, Child Mind Institute, New York, NY, United States; ^4^Center for Biomedical Imaging and Neuromodulation, Nathan Kline Institute for Psychiatric Research, Orangeburg, NY, United States; ^5^Department of Psychiatry, New York University Grossman School of Medicine, New York, NY, United States; ^6^Departamento de Psiquiatría y Psicología Clínica, Clínica Universidad de Navarra, Madrid, Spain; ^7^Departamento de Radiología, Clínica Universidad de Navarra, Pamplona, Spain; ^8^Louis A. Faillace, MD, Department of Psychiatry and Behavioral Sciences, McGovern Medical School, The University of Texas Health Science Center at Houston, Houston, TX, United States

**Keywords:** attention-deficit/hyperactivity disorder (ADHD), resting state, fMRI, stimulants, functional connectivity, reproducibility, feasibility, open science

## Abstract

Neuroimaging research seeks to identify biomarkers to improve the diagnosis, prognosis, and treatment of attention-deficit/hyperactivity disorder (ADHD), although clinical translation of findings remains distant. Resting-state functional magnetic resonance imaging (R-fMRI) is increasingly being used to characterize functional connectivity in the brain. Despite mixed results to date and multiple methodological challenges, dominant hypotheses implicate hyperconnectivity across brain networks in patients with ADHD, which could be the target of pharmacological treatments. We describe the experience and results of the Clínica Universidad de Navarra (Spain) Metilfenidato (CUNMET) pilot study. CUNMET tested the feasibility of identifying R-fMRI markers of clinical response in children with ADHD undergoing naturalistical pharmacological treatments. We analyzed cross-sectional data from 56 patients with ADHD (18 treated with methylphenidate, 18 treated with lisdexamfetamine, and 20 treatment-naive patients). Standard preprocessing and statistical analyses with attention to control for head motion and correction for multiple comparisons were performed. The only results that survived correction were noted in contrasts of children who responded clinically to lisdexamfetamine after long-term treatment vs. treatment-naive patients. In these children, we observed stronger negative correlations (anticorrelations) across nodes in six brain networks, which is consistent with higher across-network functional segregation in patients treated with lisdexamfetamine, i.e., less inter-network interference than in treatment-naive patients. We also note the lessons learned, which could help those pursuing clinically relevant multidisciplinary research in ADHD en route to eventual personalized medicine. To advance reproducible open science, our report is accompanied with links providing access to our data and analytic scripts.

## Introduction

Attention-deficit/hyperactivity disorder (ADHD) is a neurodevelopmental disorder affecting 5–10% of children and adolescents (1) and persisting into adulthood in about half of cases (2). Pharmacological treatments address the core symptoms of ADHD; long-term benefits in functional outcomes, though questioned, are documented (3). However, predicting which medication will show the best efficacy and tolerability for any given individual is not feasible. Thus, current medication management consists of trial and error titration (4).

Neuroimaging research in mental disorders, including ADHD, aims to elucidate the pathophysiology and neuropsychopharmacology of these conditions and their treatments in a quest for personalized medicine (5). Yet, despite a growing literature and increasing methodological sophistication, neuroimaging is still unable to inform “bedside” clinical decisions (6).

Resting-state functional magnetic resonance imaging (R-fMRI) has become a mainstream brain imaging technique. It highlights the statistical properties of spontaneous fluctuations in blood oxygen level dependent (BOLD) signals to infer “functional connectivity” among spatially distant areas which represent putative brain functional networks (7).

R-fMRI research in ADHD has identified widespread brain circuitry differences between patients and typically-developing control individuals (TDCs) and their possible normalization with medications; however, results have been inconsistent across studies (8, 9). Challenges to the validity and reproducibility of neuroimaging findings include insufficient control of in-scanner head motion, excessive false positive rates from inadequate control for multiple statistical comparisons, small sample size, lack of thorough reporting of methods and results and lack of open sharing of study data (10). Furthermore, research in neuroimaging and medication in ADHD has frequently used experimental treatment designs which are less relevant to finding the brain correlates of real-life treatment patterns (9).

## The CUNMET Study

The “Clínica Universidad de Navarra Metilfenidato” (CUNMET) study was designed as a proof-of-concept study in partial fulfillment of the requirements for a Ph.D. [see also (6, 9)]. The study was based at the child and adolescent psychiatry outpatient service at Clínica Universidad de Navarra, a tertiary university hospital in the north of Spain.

CUNMET's overarching objective was to evaluate the feasibility of conducting a naturalistic neuroimaging study with a clinical sample of children and adolescents with ADHD, with the aim of exploring putative R-fMRI correlates of differential symptomatic response to stimulant medications. Specific objectives were: (1) To obtain phenotypic (i.e., demographic, clinical), neuropsychological, and R-fMRI data from a cross-sectional sample of children and adolescents with ADHD with differential pharmacological responses to stimulants and a longitudinal subsample of treatment-naive patients evaluated pre- and post-treatment; (2) to conduct rigorous and transparent methods to minimize the effects of imaging artifacts, in particular, head motion and false-positive rates, in the exploration of neural correlates of treatment response; (3) to explore reliable R-fMRI differences in whole-brain network correlations across treatment-response groups and treatment-naive patients, as well as the modulation of these correlations after treatment with methylphenidate in treatment-naive patients; (4) to transparently describe the challenges encountered and the limitations of the study; and (5) to contribute to open-science efforts through best practices in reporting and data sharing.

Detailed information on methods is available in Supplementary Material. Our study included boys and girls aged 7–17 years with a diagnosis of ADHD based on DSM-5 criteria (11), falling into one of four groups: (1) patients who responded well to an extended-release formulation of methylphenidate (MPH) as the first-line treatment and were taking MPH; (2) patients who had not responded to MPH and responded to extended-release lisdexamfetamine (LDX) as second-line treatment and were taking LDX; (3) patients who had not responded to either MPH or LDX and responded to extended-release guanfacine (GFC) as the third treatment; and (4) patients who had not started medications when recruited (NAIVE). A subset of patients in the NAIVE group were invited into a longitudinal prospective pre- vs. post-treatment analysis after undergoing treatment with MPH. Clinical response was defined by either a reduction of at least 30% in parent-reported ADHD rating scale (12) or substantial improvement in Clinical Global Impression (13) after at least 3 months of the corresponding treatment. Main exclusion criteria were diagnosis of comorbid neuropsychiatric disorders and/or use of medication for symptoms of such disorders (with the exception of oppositional defiant disorder, headaches, and insomnia) and contraindications for MRI. The study had the corresponding ethical approvals, and participation was voluntary and required a written informed consent from the parents and assent from the patients.

After consent and assent were obtained along with clinical assessments that confirmed inclusion/exclusion criteria were met, R-fMRI data were acquired with a Siemens MAGNETOM 3.0 Tesla Skyra (Siemens; Erlangen, Germany) with a Siemens 32-channel head coil. Each scan session lasted around 20 min and consisted of a R-fMRI echo planar imaging BOLD sequence (total duration = 8.41 min, eyes open, TR = 2,020 ms, TE = 30 ms, 36 slices, voxel size = 3 × 3 × 3.5 mm, Field of View = 192 mm, flip angle = 80°, 250 volumes, matrix = 64 × 64), a perfusion-weighted ASL sequence (not further described here) and an anatomical T1-weighted magnetization-prepared rapid gradient-echo sequence (total duration = 5:12 min, TR = 2,300 ms, TE = 2.96 ms, number of blocks = 1, voxel size = 1.0 × 1.0 × 1.1 mm, field of view = 256 mm, flip angle = 9°, slices per block = 176, imaging matrix = 256 × 256).

Briefly, preprocessing and quality control of images used the Configurable Pipeline for Analysis of Connectomes (C-PAC, v.1.6.2a) (14) which included skullstripping, segmentation, spatial normalization of anatomical images, slice timing correction, functional-to-anatomical registration, spatial normalization, nuisance signal correction (with CompCor (15), ICA-AROMA (16) and scrubbing of volumes with more than 0.3 mm of framewise displacement, independent component analysis denoising, and median angle correction), band-pass filtering (0.01–0.1 Hz), spatial smoothing [full width at half maximum (FWHM) Gaussian kernel of 6 mm], and Z-scoring of functional image time series.

Analyses were conducted at two levels of spatial resolution. Dual regression of the Smith et al. 10-network parcellation (medial visual, occipital pole visual, lateral visual, default mode, cerebellum, sensorimotor, auditory, executive control, and right and left frontoparietal) (17) were used to extract time series of preprocessed data; we also used regions-of-interest from the Schaefer et al. 200-node parcellation (18) which overlap with the Yeo et al. 17-network parcellation (visual A&B, somatomotor A&B, temporal-parietal, dorsal attention A&B, salience-ventral attention A&B, frontoparietal control A-C, default mode A-C, limbic A&B) (19). Connectivity graphs were calculated for each individual and later used for group statistical analyses.

Descriptive and comparative statistics of relevant phenotypic data consisted of across-group tests, conducted with STATA v.12.0 (StataCorp. 2011. *Stata Statistical Software: Release 12*. College Station, TX: StataCorp LP., https://www.stata.com). Group comparisons of neuroimaging graphs were performed using the Network-Based Statistic (NBS) Toolbox (https://sites.google.com/site/bctnet/comparison/nbs) (20). Multiple comparisons were corrected through False Discovery Rate (FDR). Statistical significance was set at *p* < 0.05, corrected for multiple comparisons. The contrasts were:

(a) Stimulant-treated vs. untreated (merged MPH + LDX groups vs. naive).
(b) MPH-treated vs. untreated (MPH vs. naive).
(c) LDX-treated vs. untreated (LDX vs. naive).
(d) MPH-treated vs. LDX-treated (MPH vs. naive).

We recruited a sample of 68 participants, who provided a total of 76 scans (21 MPH patients, 21 LDX, 3 GFC, and 23 naive; 8 Treatment-naive patients were scanned pre- and post-MPH treatment). This distribution deviated from the original plan (*N* = 80; 20/group): first, GFC participants were difficult to recruit, due to the relative paucity of non-responders to stimulants in our clinic and their higher likelihood of presenting exclusion criteria; second, we were only able to obtain a post-treatment scan and evaluation in eight naive patients prior to the termination of the study at the end of the principal investigator's residency. Due to insufficient recruitment, the three GFC participants were excluded from analyses. Similarly, the eight post-treatment scans from the subgroup of naive participants who were assessed pre- and post-MPH were also excluded; instead, resources were shifted to slightly increase the samples in the MPH, LDX, and naive groups. Difficulties encountered in the recruitment process, in general, were: (a) logistical, such as scheduling issues for MRI scans due to limited scanner availability and scheduling difficulties for patients and families, many of whom lived far from the hospital; (b) constraints related to the research design, such as exclusion criteria and the challenges of complying with complex procedures on the day of scanning.

In addition, 9 participants were excluded from analyses after image quality control (7 due to excessive head motion, 1 due to a marked acquisition artifact, and 1 due to large anatomical anomalies). This led to a final sample of 56 retained participants, relatively evenly distributed across the MPH, LDX, and naive groups.

Table 1 shows the key sociodemographic and clinical characteristics of the analyzed sample, and two quality control metrics: head motion data and amount of imaging data removed per group.

**Table 1:** CUNMET included sample - key phenotypic characteristics.

**Group**	**MPH**	**LDX**	**Naive**	**Total**	**F/X^**2**^**	***p***
n included (*n* excluded)	18 (3)	18 (3)	20 (3)	56 (9)		
Age (SD) in years	14.4 (2.9)	13.3 (2.9)	12.3 (2.3)	13.3 (2.8)	2.87	0.06
Range	8.2–17.97	9.0–17.93	7.4–16.4	7.4–17.97		
Sex: boys/girls (*n*)	8/10	13/5	14/6	35/21	3.7	0.16
ADHD presentation:Combined/inattentive	10/8	12/6	10/10	32/24	1.1	0.58
Handedness^X^ (*n* = 42): Right/left handed	9/3	13/0	14/3	36/6	3.45	0.18
Comorbid ODD, *n* (%)	1 (5.6)	3 (16.7)	1 (5)	5 (8.9)	1.95	0.38
Median treatment duration in months	25	22.5	N/A	23.5	0.315	0.57
Range	3–120	6–59		3–120		
Mean framewise displacement pre-scrubbing in mm	0.089 (0.059)	0.08 (0.042)	0.09 (0.057)	0.087 (0.053)	0.2	0.82
%Retained BOLD volumes after scrubbing /total 250 (SD)	89.7% (10.5)	89.7% (10.5)	86.4% (13.8)	88.5% (11.7)	0.51	0.60

*Table of key phenotypic (sociodemographic, clinical) and imaging quality control characteristics across groups, in the sample of participants included in the imaging analyses. Results are means (with standard deviations) except for absolute numbers (n), ranges and proportions (%). Statistical differences among groups are given with their corresponding statistical test value [ANOVA F for comparison of means, Pearson's Chi-square (X^2^) for comparison of proportions and Kruskal-Wallis's Chi-square for comparison of medians] and p-values. P-values are rounded to two decimals. For continuous variables, we conducted descriptive and comparative analyses of means and ranks with both ANOVA and Kruskal-Wallis tests, as the groups were small and heterogeneous. As results were similar, ANOVA results are presented for simplicity. Framewise displacement (FD) measures head motion. Intellectual Quotients (IQ) reported here are for the full-scale*.

*A full table with all the relevant recorded data can be found on Supplementary Material*.

X *Variables with missing data*.

*MPH, group 1, on methylphenidate; LDX, group 2, on lisdexamfetamine; ADHD, Attention-deficit/hyperactivity disorder; 15q, Edinburgh Handedness Questionnaire, 15-item version; ADHD-RS.es, ADHD rating scale, Spanish version; ODD, oppositional-defiant disorder; N/A, not applicable; SD, standard deviation*.

Across-group comparisons of functional connectivity between pairs of the ten brain networks extracted through dual regression based on Smith et al. (17) yielded no statistically significant results after adjustment for multiple statistical comparisons.

**Figure 1:**
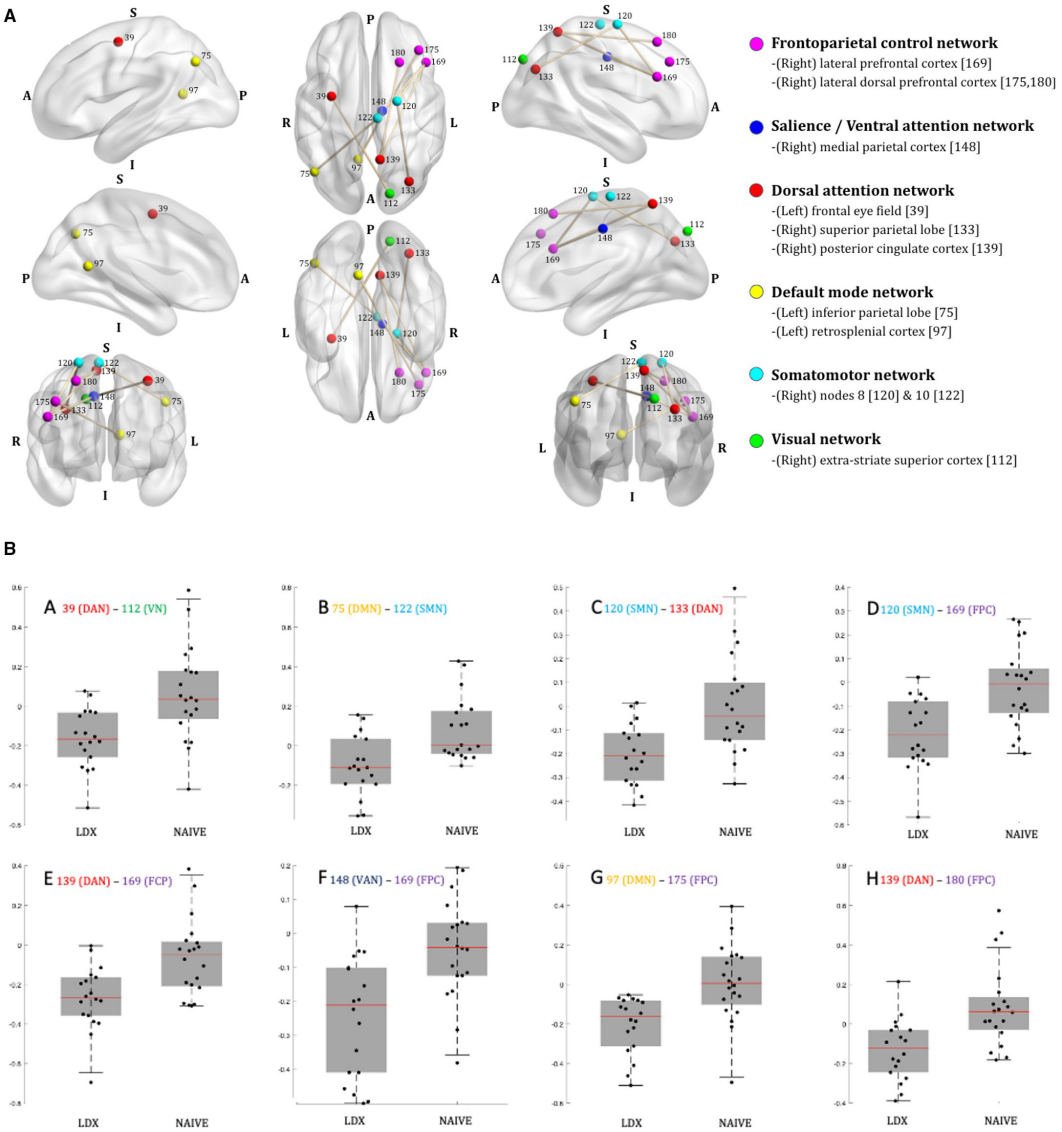
CUNMET neuroimaging results. **(A)** Projections of the hubs and edges of the pairs of brain nodes [numbered per the Schaefer et al. (18) atlas] that differed significantly on at least 5% of simulations between children treated with lisdexamfetamine vs. medication-naive patients overlaid on semi-transparent brains. The correspondence between Schaefer's numbers and node names, as well as between the colors of the edges (nodes) and the brain network they belong to [per Yeo et al. (19) brain atlas] are indicated to the right. A, anterior; P, posterior; L, left; R, right; S, superior; I, inferior. For correspondence between the Schaefer nodes and Montreal Neurological Institute brain coordinates see Supplementary Material. Graphs generated with BrainNet Viewer software (https://journals.plos.org/plosone/article?id=10.1371/journal.pone.0068910). **(B)** Box plots of between-group differences in average functional connectivity across the eight pairs of brain nodes [numbered per Schaefer et al. (18)]. LDX = study group 2, patients treated with lisdexamfetamine; naive = study group 4, treatment naive-patients. MPH group (group 1) is not presented here as it did not yield reliable statistically-significant differences in comparisons with LDX and naive, although its average functional connectivities were similar to the connectivities in the naive group. The corresponding brain networks for each node [as per Yeo et al. (19) brain atlas] are indicated by the color codes provided in the right side of **(A)**. DAN (red) = Dorsal attention network; VN (green) = Visual network; DMN (yellow) = Default mode network; SMN (light blue) = Somatomotor network; FCP (purple-fuchsia) = Frontoparietal control network; VAN (dark blue) = Salience/Ventral attention network. Graphs generated with MATLAB.

Across-group comparisons of functional connectivity between pairs of the 200 brain nodes extracted based on Schaefer et al. (18) detected 30 pairs of functional connectivity which exceeded the 5% FDR threshold in at least one of the 859 simulations. All of these occurred exclusively in the contrast between LDX and NAIVE groups. Assuming that many of these nominally significant results likely represent false positives, we focus on the eight correlations that emerged in at least 5% of the simulations. Figure 1 displays the 12 brain nodes involved in these eight pairs. They represented 10 distinct brain regions, identified with their corresponding Yeo et al. (19) networks. The nodes were predominantly located in the right hemisphere and mainly involved the frontoparietal control, attention, and default mode networks, although the somatomotor and visual networks were also represented. The box plots of average functional connectivity show that the LDX group had lower average functional connectivity in each pair than the NAIVE group; specifically, while the average functional connectivity values in these eight pairs in the NAIVE group were around zero, they were negative (i.e., anticorrelated) in the LDX group. Detailed information on results is available in Supplementary Material.

## Discussion

In this exploratory study primarily designed to assess feasibility, within the constraints of a training program and with limited resources, we confirmed that it is feasible to conduct a complex brain imaging effort within a naturalistic clinical practice setting, albeit with multiple limitations. Conducting this study was an explicit learning process. The research team was aware of the main methodological threats of R-fMRI research: small sample sizes, poor data quality due to signal artifacts [in particular, head motion (21), which is greater in children with ADHD], high false positive rates [reflecting insufficiently stringent correction for multiple comparisons (22)], excessive analytic flexibility (23), and lack of replicability of published research due to deficiencies in reporting (24). As the field has started to confront these problems, best practices in study reporting and transparency have been developed (25). In response, publicly-funded multicenter endeavors [such as ENIGMA (26) and ABCD (27)] are creating large sample sizes. In this admittedly small pilot study, we opted for stringent quality control (especially in terms of head motion corrections and exclusions), conservative statistical analyses, and transparent reporting.

Given the limitations of this study, all of our results have to be considered preliminary. In particular, we cannot be confident regarding the null results in the comparisons involving the MPH group. The greater similarity in functional connectivity patterns between the MPH group and the NAIVE group than the LDX group is surprising, as the neuropharmacological and clinical effects of MPH and LDX, both stimulants, are similar. We note a lack of R-fMRI research comparing methylphenidate vs. amphetamines (9) so cannot comment further.

Our preliminary results showed strengthened negative correlations (anticorrelations) across pairs of brain regions corresponding to different networks in children with ADHD who responded clinically to LDX after long-term treatment, when contrasted to treatment-naive patients. This finding is consistent with higher across-network functional segregation in patients treated with LDX, suggesting that distinct brain networks were operating with less interference from other networks than in treatment-naive patients. Such an increase in across-network functional segregation could be a neural correlate of positive clinical response to LDX in patients with ADHD. Due to the cross-sectional design and the lack of a healthy comparison sample, we cannot ascertain whether these differences between groups reflected pre-post medication changes, were markers of medication intake, mediators between medication intake and clinical response, or rather adaptive changes after clinical response.

The overall tentative findings, involving diverse combinations of nodes from six different networks, are compatible with systems neuroscience approaches that highlight the role of functional network segregation in health and disease. In these models, increasing segregation reflects efficient network functioning and excessive integration can be a correlate of brain dysfunction (28). If excessive cross-network functional integration were confirmed to be a consistent feature of ADHD and related neuropsychiatric disorders, it could represent a therapeutic target of medications.

## Lessons Learned

In terms of study design, the naturalistic approach, further constrained by difficulties encountered during its execution, has a limited ability to differentiate true pharmacological effects from other potential confounders, and in this sense is inferior to randomized controlled trials (9). However, this design is better suited for examining “real world” treatment responses. In addition, our cross-sectional analyses did not allow us to confirm whether across-group differences reflected true medication-induced changes. Finally, the lack of TDC individuals prevented us from formulating any tentative conclusions about the potential “normalizing” effect of medications reported in some previous studies (9) (i.e., disappearance of neuroimaging statistical differences between patients with ADHD and TDC when patients are on treatment). Nevertheless, as noted elsewhere, claims of “normalization” should be considered with caution (9).

Another major limitation was the limited sample size, even if it was in line with that of most previous studies (9). Small sizes have been endemic in neuroimaging research of ADHD (6, 8), and medication studies present further logistical complications for recruitment and retention of participants. This is partially offset by the within-subject analyses which increase statistical power relative to cross-sectional designs (29).

We adopted a naturalistic design with relatively broad inclusion criteria reflecting the patients with ADHD seen in our clinic. Thus, we recruited a sample encompassing a wide age range (7–17 years), both sexes, right and left-handed individuals, and allowed certain comorbidities (learning disabilities, oppositional defiant disorder, anxiety and mood symptoms and sub-diagnostic threshold autistic traits). This approach contrasts with previous studies which had narrower age ranges and/or excluded females (9). The down-side of our more inclusive approach is greater heterogeneity, which also decreases statistical power.

Other study limitations included inconsistencies in demographic and clinical data collected, the specific naturalistic medication scheme (which reflected clinical practice in one center), lack of mock-scanning training sessions, lack of real time monitoring of wakefulness and head motion, and flexibility of analytic pipelines (outlined in Supplementary Material) due to the exploratory nature of this study and PhD candidate training purposes.

The analytic approach was robust to limit the effects of head motion and false-positive rates, two key issues in neuroimaging research of ADHD (9), and the whole-brain network-based analyses afforded a big-picture perspective of brain functioning that transcends the study of isolated regions or networks. The statistically-salient results, along with the statistical uncertainty of their robustness, are transparently reported.

Our experience conducting the CUNMET study, with its successes, shortcomings, and preliminary results may be relevant for the design of future research in this area. We provide specific recommendations in five domains:

1. *Study design:* Design prospective naturalistic studies to assess pre vs. post-treatment changes in medication-naive patients with ADHD, optimally with a comparison group of TDC individuals scanned twice. Account for the potential effects of age/development on treatment responses and its brain correlates (30). Sample sizes of at least 50/group would improve statistical power and reproducibility (29).
2. *Participant assessment and data collection:* Ensure systematic collection of clinical, sociodemographic, and neuropsychological data in naturalistic settings. Use standard clinical assessments in initial visits and follow-ups. Conduct a thorough medication history for each patient.
3. *Image acquisition:* Include training (mock) scanning sessions to habituate participants to the experience. Optimize head immobilization tools. Monitor real-time wakefulness and head motion. Record any incidents during the session. Increase the total duration of BOLD sequence data to at least 25 min (31), by combining movie watching or tasks.
4. *Imaging and statistical analyses:* Conduct thorough quality control soon after obtaining each image. Perform rigorous nuisance and head motion corrections, and include whole-brain metrics. Consistently correct for multiple comparisons. Discuss the rationale and potential limitations of the selected methods.
5. *Transparency and reporting, and open science:* Prospectively register the study protocol, including the data analysis plan, in online platforms such as the Open Science Framework (www.osf.io), and report relevant changes from the initial protocol. Follow the Organization for the Human Brain Mapping - Best Practices in data analysis and sharing in neuroimaging using MRI (OHBM-COBIDAS) (25) to thoroughly report methods and results. Ask prospectively for permission from participants to share their deidentified data with the scientific community. Share deidentified data and full analytic scripts in online repositories.

In conclusion, the CUNMET study constituted a fruitful proof-of-concept of state-of-the-art neuropsychopharmacological research in ADHD. Despite substantial shortcomings, we demonstrated the feasibility of a naturalistic design within the constraints of real-time clinical practice and a training program, and our findings suggest that potential brain correlates of clinical response to lisdexamfetamine in children and adolescents are based in across-network segregation. The lessons learned from CUNMET are offered to inform future research and to encourage prospective investigators to undertake transparent, open-science collaborative efforts. While similar individual small studies will not advance the field on their own, their aggregation may offer frameworks for efficient exploration of designs, methods, and potential biomarkers (32) in the quest for personalized medicine in ADHD.

## Data Availability Statement

The datasets presented in this study can be found in online repositories. The names of the repository/repositories and accession number(s) can be found below: The CUNMET Study team supports open science and is sharing de-identified patient data and analytic scripts to allow other researchers to carry out independent analyses, providing they acknowledge the source of the data and its funding in their reports. Deidentified and defaced brain imaging and phenotypic data from the patients for whom we received permission for sharing are available at INDI (http://fcon_1000.projects.nitrc.org/indi/retro/CUNMET.html). Our analytic codes are available at GitHub (https://github.com/victorpsanchez/cunmetstudy).

## Ethics Statement

The CUNMET study was registered by the Spanish Agency of Drugs and Medical Products on March 27, 2017, with the registration code CUN-MET-2017-01. The statistical group analysis plan was registered at Open Science Framework (https://osf.io/2dfs8) on September 24, 2019, after data collection and pilot preprocessing and before conducting group analysis. The study was ethically reviewed and approved by the Ethics Committee for Medications Research of Navarra (Spain) (Comité de Ética de la Investigación con medicamentos, CEIm de Navarra) on June 21, 2017, with the codes CUNMET-2017-01 EO17/11, which also approved an amended protocol on May 22, 2019. This study was compliant with the research ethics principles of the Declaration of Helsinki (seventh revision, 2013), taking into account the specific principles for research with children and adolescents. Written informed consent to participate in this study was provided by the participants' legal guardian/next of kin.

## Author Contributions

VP-S led the study design, data collection and analysis, reporting of results, and prepared the manuscript. AF assisted with data analysis, reporting of results, and manuscript preparation. PC-M and CS supervised the study design, data collection, and reporting of results, and reviewed the manuscript. MF-S, MV-V, AD-S, and MG assisted in the study design, data collection and analysis, and reviewed the manuscript. MF-M assisted in the data analysis and reviewed the manuscript. MM assisted in the study design, supervised the data analysis, and reviewed the manuscript. FC supervised the study design, data collection and analysis, reporting of results, and reviewed the manuscript. All authors approved the final version of the manuscript and assumed their responsibility as coauthors.

## Funding

The research procedures of the CUNMET study were supported by private funds from the Department of Psychiatry and Medical Psychology, Clínica Universidad de Navarra (Fondo de Reservas N.2222, PI PC-M). The costs of the gift cards given to the participants were provided by a research-supporting foundation which preferred to remain anonymous. VP-S's training position and the article processing fees for this publication were covered by Fundación Alicia Koplowitz.

## Conflict of Interest

The authors declare that the research was conducted in the absence of any commercial or financial relationships that could be construed as a potential conflict of interest.

## Publisher's Note

All claims expressed in this article are solely those of the authors and do not necessarily represent those of their affiliated organizations, or those of the publisher, the editors and the reviewers. Any product that may be evaluated in this article, or claim that may be made by its manufacturer, is not guaranteed or endorsed by the publisher.
