# Supplementary material for: Resting-State fMRI to Identify the Brain Correlates of Treatment Response to Medications in Children and Adolescents With Attention-Deficit/Hyperactivity Disorder: Lessons From the CUNMET Study
Source: Front Psychiatry. 2021 Nov 16;12:759696. doi: 10.3389/fpsyt.2021.759696 (PMC8635006; doi:10.3389/fpsyt.2021.759696)
Supplement: Supplementary file 1 [file Data_Sheet_1.pdf]

SUPPLEMENTARY MATERIAL

Expanded Hypotheses and Objectives

Hypotheses

From a systems neuroscience perspective, the default mode network (DMN) interference hypothesis (1), posits that ADHD reflects aberrant cross-network interference, especially between the DMN and task-positive networks. Apart from the specific putative role of the DMN in the pathophysiology of ADHD, previous research has also pointed out at more generalized across-network integration as a possible hallmark of whole-brain network disruptions in patients with ADHD (2). These patterns should be most evident in medication-naive children with ADHD, relative to those being treated effectively. Such effective therapeutic effects, such as by stimulants, should produce 'normalization,' i.e., greater segregation between DMN and task-positive networks. Further, the degree of therapeutic improvement should be related to the extent of increased inter-network segregation. This study originally included children being treated with guanfacine to provide a contrast of another pharmacologic agent that has some evidence of efficacy but through alternative mechanisms, to consider whether the broad effects at the level of large-scale networks would parallel or diverge from those of the stimulants. Finally, this study included children with the two principal types of stimulants, methylphenidate and amphetamine, to also explore whether their neuroimaging correlates would mostly overlap or diverge.

The overarching question was whether a naturalistic approach, which has the advantage of feasibility and potential scalability, could provide insights into therapeutic mechanisms of action. If so, a model of aggregating such samples from multiple clinical settings could advance towards the ultimate goal of personalized medicine through the incorporation of biomarkers into clinical practice (3) to improve the care of children and adolescents with ADHD.

Objectives

A. Overarching objective:

The overarching objective of the Clinica Universidad de Navarra Methylphenidate Treatment (CUNMET) project was to deploy a neuroimaging study with a naturalistic clinical sample of children and adolescents with ADHD, to evaluate the feasibility of a research design aimed at exploring putative R-fMRI correlates of differential symptomatic response to stimulant medications.

B. Specific objectives:

1. To obtain phenotypic (i.e., demographic, clinical), neuropsychological, and R-fMRI data from a cross-sectional sample of children and adolescents with ADHD with differential pharmacological responses to stimulants (patients who responded to methylphenidate, patients who did not respond well to methylphenidate but responded to lisdexamfetamine, and patients who did not respond to either stimulant but responded to guanfacine) and a longitudinal sample of treatment-naive patients evaluated pre- and post-treatment.

2. To conduct rigorous and transparent methods to minimize the effects of imaging artifacts, in particular, head motion, and false-positive rates, in the exploration of neural correlates of treatment response.

3. To explore reliable R-fMRI differences in whole-brain network correlations across treatment-response groups and treatment-naive patients, as well as the modulation of these correlations after treatment with methylphenidate in treatment-naive patients.

4. To transparently describe the challenges encountered and the limitations of the study.

5. To contribute to open-science efforts through best practices in reporting and data sharing.

Expanded Methods

Figure S1 presents a flowchart of the study procedures, described thoroughly below.

Figure S1: Flowchart of study procedures

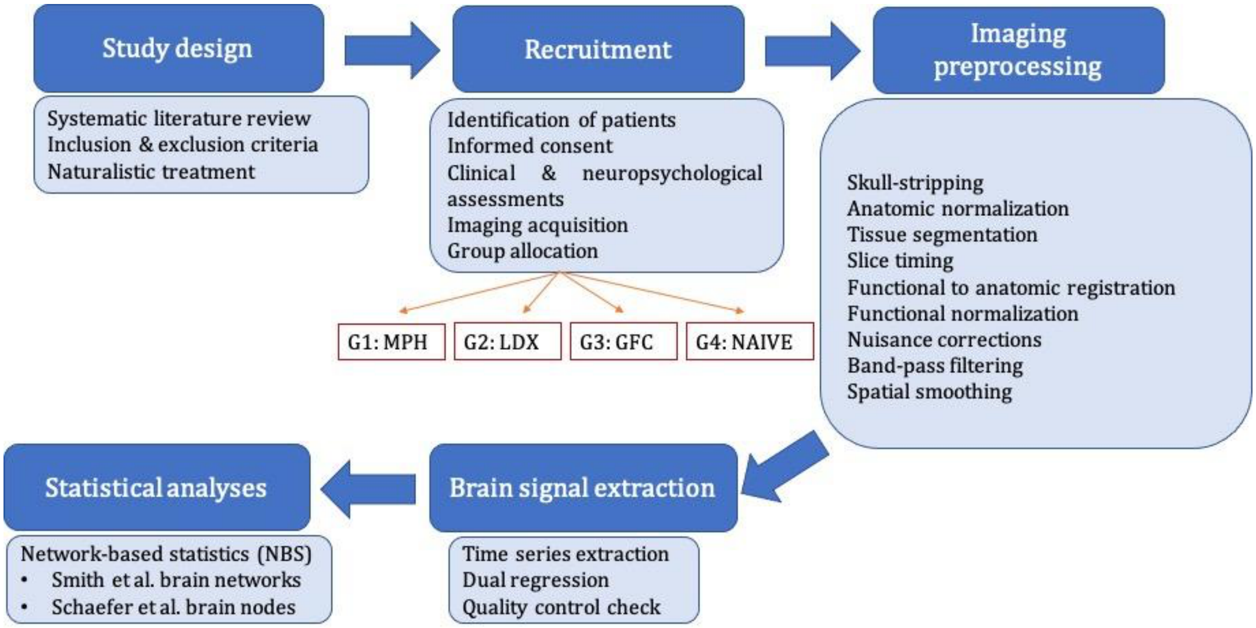

The study design was followed by patients' recruitment and allocation into groups 1 (G1: MPH, patients with good response to methylphenidate), 2 (G2: LDX, patients not responsive to methylphenidate, and with good response to lisdexamfetamine), 3 (G3: GFC, patients not responsive to stimulants, and treated with guanfacine), and 4 (treatment-naive patients, of which some underwent pre- and post-medication assessments). Acquired R-fMRI images were preprocessed, and brain signals were extracted and checked for quality. Finally, statistical across-group analyses for R-fMRI signals on the Smith et al. brain networks (4) and the Schaefer et al. brain nodes (5) were conducted with network-based statistics (NBS) (6).

Study design

The CUNMET-BOLD observational study involved a cross-sectional analysis and a pilot prospective exploration of resting-state intrinsic brain activity metrics obtained with fMRI BOLD in the CUNMET cohort.

The CUNMET project started in 2016 at the Department of Psychiatry and Medical Psychology, University of Navarra Clinic (*Clínica Universidad de Navarra* (CUN)), a tertiary university hospital in Pamplona, Navarra, Spain, founded in 1952, that since 2017 also has a site in Madrid (400 km away). Its goal was to explore potential neural correlates of treatment response in children

and adolescents with ADHD in a clinical outpatient setting. It involved the retrospective, prospective, and cross-sectional collection of phenotypical (sociodemographic, clinical, neuropsychological), structural and functional neuroimaging (BOLD-based resting-state fMRI) and blood-sample (serum ferritin and serum methylphenidate levels) data from pediatric patients with ADHD treated naturalistically with medications. Although not a part of this report, arterial spin labeling (ASL) data were also obtained to quantify perfusion. These data will be made openly available to the scientific community along with the other modalities obtained.

The design was grounded in a literature review and longitudinally refined with the results of our systematic review of previous studies using R-fMRI to assess the neural correlates of medications for ADHD (7).

Participants

Study participants were children and adolescents in an outpatient clinical setting at the University of Navarra Clinic Child and Adolescent Psychiatry Unit, Outpatient program. The study design included naturalistic treatment provided by the corresponding child psychiatrists in accordance with their clinical judgment.

<u>Inclusion criteria</u> were:

(1) Children and adolescents with ages between 7-17 years (inclusive).
(2) Correct communication in Spanish.
(3) Under evaluation or follow-up at the outpatient Clinics of the Department of Psychiatry and Medical Psychology, Child & Adolescent Psychiatry Unit.
(4) Diagnosed with DSM-5 (8) ADHD, any current presentation, after a general diagnostic interview supervised by CUN child and adolescent psychiatrists.
(5) Able to undergo MRI scans and neuropsychological tests.
(6) Provide explicit written consent from parent/s or legal guardian/s (in adolescents between ages 12-17 years inclusive, an explicit oral or written assent was additionally required).
(7) Adherence to the pharmacological treatment prescribed for their ADHD, if currently under treatment.
(8) Falling into one of the four study groups, according to pharmacological treatment response to study medications (methylphenidate, lisdexamfetamine, guanfacine, or naive).

<u>Exclusion criteria</u> were:

(1) Past or co-occurring DSM-5 diagnosis of any psychiatric disorder with the exception of oppositional-defiant disorder (ODD) and past anxiety disorder with current remission or stable symptoms,
(2) diagnosis of other relevant central nervous system (CNS) disorders, diseases, or malformations (including diseases such as epilepsy, but not including headaches or insomnia),
(3) co-occurring or past long-lasting (for more than a year) or recent (during the last 6 months) lifetime exposure to any CNS drug with the exception of ADHD drugs for groups 1-3, melatonin, short/medium half-life anxiolytic/hypnotics (taken at night and at least 12 hours prior to the MRI scan) and treatments for headaches,

(4) current clinically-reported frequent use or lifetime abuse of alcohol, cannabis, and illegal drugs,
(5) severe claustrophobia and
(8) having irremovable metals in the head (including orthodontic appliances (9,10)) or any other devices in the body incompatible with an MRI scan (e.g., pacemakers).

Treatment-response criteria

Positive treatment response, based on previous treatment response research in ADHD (11–13), was defined as meeting either of the following two criteria after at least one month of treatment with study medications at an appropriate dose:

(a) Reduction of ≥30% in parent-reported ADHD-RS.es (ADHD Rating scale from DuPaul et al. (14), translated into Spanish and validated on a local sample at our department (15);
(b) final CGI-I (Clinical Global Impression-Improvement) (16,17) of 1 ("very much improved") or 2 ("much improved"), scored directly by the attending clinician, or retrospectively estimated based on clinical notes.

Change of medications due to adverse effects was not considered a criterion of lack of response *per se* unless it was associated with a lack of clinical efficacy.

Study groups

Depending on the treatment response to study medications, the following groups of patients were defined:

(a) Group 1 (MPH group) was composed of patients who were good responders to an extended-release formulation of methylphenidate (Concerta®, Equasym®, or Medikinet®) as the first-line treatment and were taking this medication at the moment of the recruitment.
(b) Group 2 (LDX group) was composed of patients who had not responded to methylphenidate as the first option and subsequently responded to an extended-release formulation of lisdexamfetamine (Elvanse®) as second-line treatment and were taking this medication at the moment of the recruitment.
(c) Group 3 (GFC group) was composed of patients who had not responded to stimulant medications (methylphenidate or lisdexamfetamine) and responded to an extended-release formulation of guanfacine (Intuniv®) as a third-line treatment.
(d) Group 4 (NAIVE group) comprised patients who had not started medications at the time of the recruitment. Some of the patients of the NAIVE group were included in a longitudinal prospective pre vs. post-treatment analysis after they began treatment with any of the aforementioned formulations, with methylphenidate as the first-line treatment.

The study aimed to recruit a total of 80 patients, 20 per group, with at least 10 patients from the NAIVE group having a pre and post-treatment fMRI scan. This sample size was estimated based on previous literature and the recruitment prospects in our center. It was also intended that the four groups would be as homogeneous as possible in terms of age, sex, and intellectual quotient (IQ), although it was not possible to precisely match these variables due to the naturalistic recruitment process, on the one hand, and inherent age-group bias, on the other, as patients taking second or third-line medications (LDX and GFC groups) tended to be older than those who responded to the first-line medication (MPH group) or who were medication-naive (NAIVE group).

However, the research team encountered difficulties in finding sufficient patients in the GFC group. After three waves of recruitment for this group, it was decided to discontinue their recruitment and remove them from imaging analyses. It was also decided to increase the other groups by 1-3 patients/each. Furthermore, it was only possible to obtain a second (post-treatment) fMRI in eight patients in the NAIVE group because of scheduling difficulties and because of completion of the principal investigator's residency training. Thus, the analysis did not include the planned contrasts of pre and post-treatment data or the GFC group.

Patient assessments and data collection
All recruited patients had undergone, as part of their initial outpatient clinic assessment, a general psychiatric diagnostic interview in accordance with DSM-5 criteria (8), conducted by a resident physician in psychiatry or a clinical psychology trainee, with supervision and diagnostic confirmation by a child and adolescent psychiatrist. Follow-up visits were conducted either by trainees supervised by specialists or by the specialists directly.

Initial assessment also included a set of qualitative and symptom-scoring questionnaires completed by patients, their parents or caregivers, and their teachers. The primary measure was the ADHD-RS.es (15), completed by parents or caregivers during the initial assessment and follow-up visits and used as the primary criterion for treatment response. Patients' parents or caregivers were asked to provide a single questionnaire integrating their common impressions.

In most cases, clinicians provided an explicit CGI-S scale score (16,17) at the initial assessment and at follow-up visits (along with post-treatment CGI-I scores in those visits). In most cases, CGI scores before and after each of the treatments were used as a secondary criterion for characterizing treatment response. When the CGI was not recorded explicitly, a retrospective estimation was made by the principal investigator based on the clinical notes taken on that appointment by the corresponding clinician; in those cases, a CGI-I was scored as 1 when the pre/post-treatment change of CGI-S was greater than 2 points and/or the post-treatment CGI-S was 1 ("Normal/Not ill").

Regarding neuropsychological assessments, the initial assessment visit routinely included the estimation of IQ scores (full scale, verbal, non-verbal, working memory and processing speed) through Wechsler-based methods (WIPPSI, WISC-IV, or WISC-5) (18) or Kaufman Brief Intelligence Test (K-BIT) (19,20), and an evaluation of Executive Function and attention using the Stroop color and word test (21) and the MOXO-Continuous Performance Test (MOXO-CPT) (22). These evaluations were conducted by a trained psychiatric nurse or clinical psychology resident competent in the use of these tests, and their results were supervised by a clinical psychologist.

Neuropsychological tests varied somewhat over time. IQ scores were routinely assessed through WISC-IV and WIPPSI until June 2015, when the K-BIT was introduced to provide full scale, verbal and non-verbal IQ; the WISC-IV was still used to obtain working memory and processing speed scores. Starting in February 2018, the WISC-5 replaced the WISC-IV for that purpose and for obtaining all IQ indices. The Stroop test did not vary, whereas the MOXO-CPT replaced a conventional CPT in September 2016. Other factors included test form availability or decisions by the evaluator or referring psychiatrist. For this study, we included the results of the most recent (in

relation to the fMRI scan) IQ evaluations (together with other tests administered at the same time) for all patients in the sample. The only Stroop and MOXO-CPT scores used were from the NAIVE group, when the tests were conducted within about a week from the date of the pre-treatment MRI scan.

Finally, two other relevant factors were considered for this study: parental education and handedness. Parental education was collected by asking about father's and mother's careers, and clustering their responses into customized categories based on the ABCD study phenotypic assessments (23) adapted by the research team to match the Spanish education system. Patient handedness was assessed with a customized Spanish translation of the 15-question Edinburgh inventory (24) (Edinburgh augmented version, here called 15q), completed by the parents or administered by clinicians to the patients directly.

A phenotypic dataset was collected with the relevant sociodemographic, neuropsychological, and clinical data. The research team has made that dataset and the MRI data, de-identified, publicly available in an open online repository for further analyses [http://fcon_1000.projects.nitrc.org/indi/retro/CUNMET.html].

MRI acquisition

Resting-state brain MR images were acquired with a whole-body Siemens MAGNETOM Skyra (Siemens; Erlangen, Germany) with a magnetic strength field of 3.0 Tesla and a 32-channel head coil, which is available for clinical and research purposes at CUN. Each scan session lasted around 20 minutes in total and consisted of initial field mapping sequences, a resting-state fMRI Echo Planar Imaging (EPI) BOLD sequence (total duration=8.41min, eyes open, TR=2020ms, TE=30ms, 36 slices, voxel size=3x3x3.5mm, Field of View=192mm, flip angle=80º, 250 volumes, matrix=64x64), a perfusion-weighted ASL sequence (not further described here) and an anatomical T1-weighted magnetization-prepared rapid gradient-echo (MPRAGE) sequence (total duration=5:12min, TR=2300ms, TE=2.96ms, n. of blocks=1, voxel size=1.0x1.0x1.1mm, field of view=256mm, flip angle=9º, slices per block=176, imaging matrix=256x256), conducted in that order.

During all the scanning sessions, patients were instructed to remain still, awake, and with eyes open without fixation on any particular point. Patients were supine and wearing a standard head coil and cushions to minimize head motion. We used a video camera to detect gross in-scanner motion, and verbal feedback was provided to remind patients to keep still if gross motion was observed. Wakefulness was not formally monitored, although scanner technicians checked on patients in between sequences, and no patient reported falling asleep during the functional sequences. All the patients underwent a scan session upon recruitment. Due to the limited availability of the scanner and school and work schedules, almost all scans were conducted during weekday afternoons or evenings, about 8-12 hours after their usual intake of medication, although the precise time of medication intake was not recorded.

All T1-weighted images were screened for abnormal findings by a clinical neuroradiologist at CUN; significant abnormalities (n=3) were reported to the parents of the patients, and these participants were excluded from the imaging analyses.

Neuroimaging preprocessing
Brain images were pulled from the scanner to the electronic health record of each patient at the IT system of our hospital, within a Picture Archiving and Communication System (PACS) environment. Images were retrieved in DICOM format, de-identified with a DICOM coder (changing the subject names and medical record identification numbers to study subject codes), converted to NIfTI format (25) using dcm2nii software (26), organized per brain imaging data structure (BIDS) standards (27), and stored in an encrypted cloud environment to be accessed by the research team.

The pipelines for preprocessing the T1-weighted and BOLD images and extraction of resting-state BOLD metrics were developed after conducting preliminary explorations of data structure and quality control on different pipelines. The research group has made the final scripts publicly available at [https://github.com/victorpsanchez/cunmetstudy].

Preprocessing pipelines were run on a high-performance supercomputer (New York University Langone Health 'Big Purple') using a Singularity (v.2.5.2) container of the Configurable Pipeline for the Analysis of Connectomes (C-PAC, v.1.6.2a) (28), an open-source software developed and maintained by the Child Mind Institute, which harnesses 3rd-party open-source tools mainly based on the Analysis of Functional NeuroImages (AFNI, v.20.1.17) (29,30) and FMRIB Software Library (FSL, v.5.0) (31). The final preprocessing pipeline included the following steps:

<u>A. Structural Images:</u>

(1) Skullstripping (i.e., removal of non-brain structures, including the skull) of the T1-weighted images with Advanced Normalization Tools (ANTS) (32) package.
(2) Anatomical segmentation of the T1-weighted images into white matter, gray matter, and cerebrospinal fluid tissues based on prior probability maps with the FSL-FAST package,
(3) Anatomical spatial normalization (i.e., registration with a standard brain atlas) of the T1-weighted images with the Montreal Neurological Institute (MNI)-152 template (33,34) using the ANTS package.

<u>B. Functional Images:</u>

(1) Slice timing correction with the AFNI-3dTshift package.
(2) Functional-to-anatomical images registration using the ANTS package.
(3) Functional to MNI registration with a 3mm resolution using the ANTS package.
(4) Nuisance signal corrections through:
    (a) Regression of motion parameters, quadratic and linear trends, noise regions of interest (ROIs; i.e., white matter and cerebrospinal fluid), and modeling of physiological noise with a 5 principal component analysis (CompCor) (35).
    (b) Realignment of the 24-parameter Friston model (36) derived from the volume realignment with AFNI-3dvolreg.
    (c) Scrubbing (censoring) of volumes with an estimated framewise displacement (FD) > 0.3mm (along with the immediately previous and posterior volumes) as calculated by the Jenkinson method (37).
    (d) Independent component analysis - automatic removal of motion artifacts (ICA-AROMA) denoising (38) through FSL-MCFLIRT.
    (e) Median angle correction (39) with a target angle of 90º.

(4) Band-pass filtering of frequencies outside the range of 0.01-0.1Hz with AFNI-3dBandpass.
(5) Spatial smoothing of the images with a full width at half maximum (FWHM) Gaussian kernel of 6mm with FSL.
(6) Z-scoring the voxel time series (removing the mean and dividing by the standard deviation).

Time series of preprocessed BOLD data were extracted through two different techniques:
(a) Dual regression (DR) - Networks based on Smith et al.'s 10 networks (4): medial visual, occipital pole visual, lateral visual, default mode, cerebellum, sensorimotor, auditory, executive control, and right and left frontoparietal networks.
(b) Regions of interest (ROI) - Time series were extracted using the Schaefer 200 parcellation (5). The Schaefer parcellation, in which brain nodes correspond to hubs in the 17 brain networks described by Yeo et al. (40) (visual A&B, somatomotor A&B, temporal-parietal, dorsal attention A&B, salience-ventral attention A&B, frontoparietal control A-C, default mode A-C, limbic A&B).

Based on these extraction techniques, connectivity graphs were calculated for each individual, by either calculating the correlations between DR networks or ROIs. These graphs were then used for group statistical analyses.

Quality control was checked for all images during and after preprocessing. This included calculation of head motion metrics and the quantity of motion-free data and a visual inspection of the outcomes of the anatomic-functional-standard space image registration. Patients were removed from the functional brain imaging metrics group analyses according to the following criteria:
(a) Excessive head motion, as defined by a mean FD>0.3mm.
(b) Acquisition artifacts ascertained visually that distorted the fMRI signal.
(c) Anatomical lesions or variants ascertained visually which distorted the fMRI signal.

Statistical analyses
Phenotypic
Descriptive and comparative statistics of relevant phenotypic (sociodemographic and clinical) data, as well as head motion parameters, consisted of across-group ANOVA/Kruskal-Wallis and chi-square tests, depending on the characteristics of the variables, conducted with STATA v.12.0 (StataCorp. 2011. *Stata Statistical Software: Release 12*. College Station, TX: StataCorp LP., https://www.stata.com).

Neuroimaging
Group comparisons of neuroimaging graphs were performed using the Network-Based Statistic (NBS) (6) Toolbox (https://sites.google.com/site/bctnet/comparison/nbs). Multiple comparisons were corrected through False Discovery Rate (FDR). Statistical significance was set at $p<0.05$, corrected for multiple comparisons. The cross-sectional group comparison contrasts were:
(a) Stimulant-treated vs. untreated (merged MPH+LDX groups vs. NAIVE group).
(b) MPH-treated vs. untreated (MPH group vs. NAIVE group).
(c) LDX-treated vs. untreated (LDX group vs. NAIVE group).
(d) MPH-treated vs. LDX-treated (MPH group vs. LDX group).

Given the stochastic nature of FDR, each statistical test was performed 859 times (limited by computer memory constraints). Pairs of regions that showed significant correlations in at least 5% of the statistical tests were identified, and within these regions, t-tests were repeated including age as a covariate.

Ethical and legal considerations and best practices
The overall CUNMET study was registered as a 'post-authorization study with a design different from a prospective follow-up' by the Spanish Agency of Drugs and Medical Products (Agencia Española de Medicamentos y Productos Sanitarios) on March 27, 2017, with the registration code CUN-MET-2017-01 (the registration protocol included details on the overarching hypotheses, goals, and methods, without preprocessing and statistical analysis details). The statistical group analysis plan was registered at Open Science Framework (https://osf.io/2dfs8) on September 24, 2019, after data collection and pilot preprocessing and before conducting group analysis.

The CUNMET study was ethically reviewed and approved by the Ethics Committee for Medications Research of Navarra (Spain) (Comité de Ética de la Investigación con medicamentos, CEIm de Navarra) on June 21, 2017, with the codes CUNMET-2017-01 EO17/11, which also approved an amended protocol on May 22, 2019. This study was compliant with the research ethics principles of the Declaration of Helsinki (seventh revision, 2013), taking into account the specific principles for research with children and adolescents.

During recruitment, potential patients and their families were given an overview of the study, including the potential risks and expected benefits of their participation, and making it clear that their involvement in the study would not affect their routine psychiatric care in our department nor their treatment prescriptions and schedules. Participants were offered a gift worth 20€ or gift card as compensation for the inconveniences related to involvement in the study. Parents agreeing to their child's participation were asked to read and sign an informed consent form, explicitly providing their consent to the fMRI scan, a blood test (if applicable, including genetic tests), and public sharing of the anonymized clinical and neuroimaging data. A separate consent form was also collected for those patients providing blood samples to be stored in an encrypted biobank at our university for later analysis.

After finishing the recruitment, data from the participants were de-identified of any personal identifiable information and stored in shared cloud files for analysis by the research group. The research team of this study, with the approval of the Ethics Committee, has made the relevant de-identified data and analytic scripts public online for the wide research community [phenotypic and neuroimaging data available here: http://fcon_1000.projects.nitrc.org/indi/retro/CUNMET.html; analytic scripts available here: https://github.com/victorpsanchez/cunmetstudy] to be reused and further explored according to current best practices of transparency and open science in medical and neuroimaging research (COBIDAS) (41). This report has followed these practices as best as possible.

Expanded Results

Feasibility, participant sample, and data quality control

Figure S2 shows the flowchart of patient recruitment and exclusion. A sample of 68 participants was finally recruited, who provided a total of 76 scans (21 patients in the MPH group, 21 in the LDX group, 3 in the GFC group, and 23 in the NAIVE group; 8 patients in the NAIVE group were scanned pre and post-medication treatment with methylphenidate). This distribution deviated from the original plan: first, participants in the GFC group were difficult to recruit, due to the relative paucity of non-responders to stimulants in our clinical setting (and in the general population (42)) and their higher likelihood of presenting exclusion criteria (comorbidities and concomitant psychopharmacological medication, such as antipsychotics); second, we were only able to obtain a post-treatment scan and evaluation in eight patients in the NAIVE group, due to logistical difficulties including the completion of the principal investigator's residency training. Due to insufficient recruitment, participants from the GFC group were excluded from analyses, as were the post-treatment scans from the subgroup of NAIVE participants who were assessed pre and post-medication; instead, the research team shifted resources to slightly increase the samples in the MPH, LDX, and NAIVE groups. Difficulties encountered in the recruitment process, in general, were: (a) logistical, such as scheduling issues for MRI scans due to limited scanner availability and scheduling difficulties for patients and families, many of whom lived far from the hospital; (b) constraints related to the research design, such as exclusion criteria and the challenges of complying with complex scanning day procedures.

In addition, 9 participants were excluded from analyses after image quality control (7 due to excessive head motion, 1 due to a marked acquisition artifact, and 1 due to large anatomical distortions that made it impossible to normalize the images into standard space). This led to a final sample of 56 retained participants, relatively evenly distributed across the MPH, LDX, and NAIVE groups.

Figure S2: Patient recruitment and exclusion flowchart

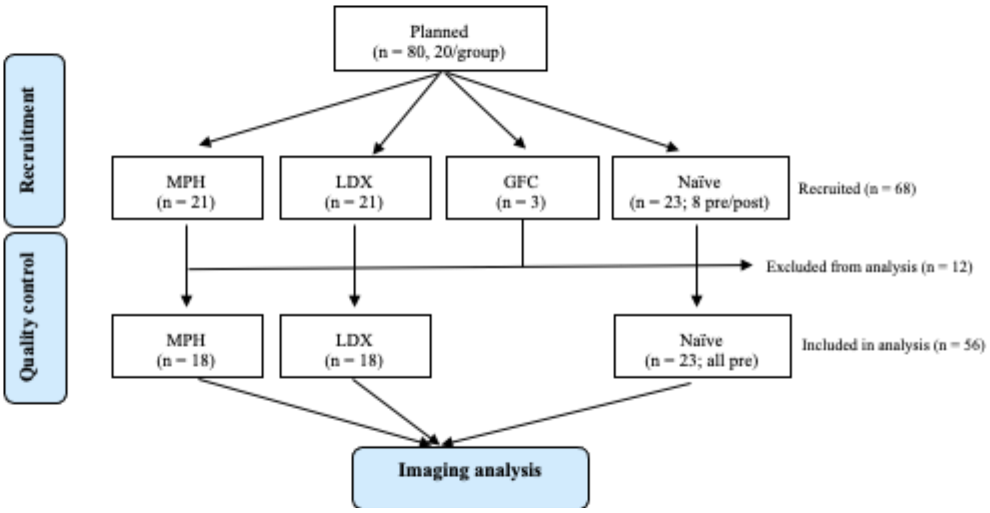

A total of 56 subjects were included in the imaging analyses, after excluding 12 due to small subsample size or quality control issues. The planned total sample of 80 (20/group) was not attained.

Table S1 shows the full sociodemographic and clinical characteristics of the participants ('final analysis sample'), and quality control metrics: head motion data and amount of imaging data removed for each group. The sample was predominantly Caucasian right-handed boys and girls with average IQ, low comorbidity, and from parents with higher education. Across-group statistically significant differences were found for processing speed (lower in the LDX group), current ADHD-RS.es rating scores and CGI-S (higher in the NAIVE group), percent symptomatic change with treatment (lower in the LDX versus the MPH group), and the age of ADHD diagnosis (lower in the LDX group). Additionally, although the statistical comparison showed a p-value slightly above the threshold for significance, it should be noted that there were differences in average age across groups of around one year, in this youngest-to-oldest order: NAIVE<LDX<MPH. Additionally, even though the across-group differences in sex proportions were not statistically significant, it is noteworthy that the whole sample and the LDX and NAIVE groups were predominantly composed of boys, while the MPH sample was more balanced in sex, with a slight female predominance. Head motion metrics and the quantity of usable imaging data were similar across groups.

Table S1: Phenotypic characteristics of patients included in the analysis

**Group**	**MPH**	**LDX**	**Naive**	Total	F/X^2	p
**N. of patients**	18	18	20	56	NA	NA
**Age (SD) in years**	14.42 (2.92)	13.28 (2.87)	12.29 (2.38)	13.29 (2.81)	2.87	0.065
**Age range**	8.16-17.97	9-17.93	7.45-16.38	7.45-17.97		
**Sex: Boys/Girls, n**	8/10	13/5	14/6	35/21	3.70	0.16
**Handedness (n=42)**						
**Edinburgh 15q scores (SD)**	+55.55 (66.48)	87.44 (12.10)	58.24 (63.38)	66.51 (54.77)	1.41	0.26
**Right/left-handed, n**	9/3	13/0	14/3	36/6	3.45	0.18
**Father's education, median (n=44)**	University degree	Vocational school / University degree	University degree	University degree	1.34	0.51
**Mother's education, median (n=44)**	University degree	Vocational school / University degree	University degree	University degree	5.37	0.068
**Ethnicity, n (%)**					7.89	0.25
**European**	17 (94.4)	16 (88.9)	17 (85.0)	50 (89.3)		
**Latin American**	1 (5.6)	0 (0)	2 (10)	3 (5.4)		
**Chinese**	0 (0)	0 (0)	1 (5)	1 (1.7)		

**Mixed**	0 (0)	2 (11.1)	0 (0)	2 (3.6)		
**Intellectual quotient (IQ)**						
**Full-scale IQ (SD)**<br>**range**	103.0 (11.6)<br>75-117	100.0 (12.4)<br>78-123	104.2 (13.3)<br>78-135	102.5 (12.4)<br>75-135	0.55	0.58
**Verbal IQ (SD)**	103.3 (12.9)	101.9 (14.8)	101.9 (14.5)	102.34 (13.9)	0.06	0.94
**Non-verbal IQ (SD)**	105.3 (12.3)	106.0 (12.2)	107.6 (10.4)	106.3 (11.5)	0.19	0.83
**Working memory (SD) (n=50)**	101.5 (15.0)	98.4 (10.9)	99.7 (15.0)	100.0 (13.9)	0.18	0.83
**Processing speed (SD) (n=50)**	108.6 (10.3)	97.2 (11.0)	106.8 (13.3)	104.9 (12.4)	3.85	0.028
**ADHD presentation, n (%)**					1.1	0.58
**Combined**	10	12	10	32		
**Inattentive**	8	6	10	24		
**Hyperactive/impulsive**	0	0	0	0		
**ADHD-RS.es scores (day of MRI scan)**						
**Total (SD)**<br>**[baseline/pre-treatment]**	11.9 (7.9)<br>32.3 (7.2)	15.9 (6.5)<br>26.7 (10.1)	31.1 (12.9)<br>NA	20.1 (12.7)<br>29.5 (9.08)	20.97<br>3.62	<0.001<br>0.066
**Inattention (SD)**<br>**[baseline/pre-treatment]**	7.7 (5.3)<br>20.5 (3.8)	9.5 (4.4)<br>15.0 (4.3)	18.4 (6.4)<br>NA	12.1 (7.2)<br>17.8 (4.8)	21.2<br>16.75	<0.001<br>0.0002
**[cont. from above] Group**	**MPH**	**LDX**	**NAIVE**	Total	F/X^2	p
**Hyperactivity/impulsivity (SD)**<br>**[baseline/pre-treatment]**	4.2 (3.6)<br>11.8 (6.8)	6.4 (4.0)<br>11.7 (6.9)	12.6 (8.8)<br>NA	8.0 (7.0)<br>11.8 (6.8)	9.97<br>0	0.0002<br>0.98
**Percent change with treatment (SD) %**	62.2 (24.0)	33.7 (37.0)	NA	47.9 (33.9)	7.5	0.0098
**CGI-S (day of MRI scan), median (range)**<br>**[baseline/pre-treatment]**	2 (1-3)<br>4 (3-5)	2 (1-5)<br>4 (3-5)	4 (3-6)<br>NA	2 (1-6)<br>4 (3-5)	37.46<br>0.66	0.0001<br>0.42
**Dose (mg/kg/day) (SD)**	1.01 (0.24)	1.12 (0.41)	NA	NA	NA	NA
**Birth weight (kg) (n=52) (SD)**	3.18 (0.67)	3.19 (0.50)	3.37 (0.51)	3.25 (0.56)	0.68	0.51

**Adopted, n (%)**	1 (5.6)	0 (0)	3 (15.0)	4 (7.1)	3.31	0.19
**Age at diagnosis (SD)**	11.0 (3.9)	8.1 (2.8)	11.4 (2.9)	10.2 (3.5)	5.53	0.0066
**Treatment duration (months), median range**	25<br>3-120	22.5<br>6-59	NA	23.5<br>3-120	1.05	0.31
**Family history of ADHD in 1st degree relatives, n (%)**	5 (27.8)	9 (50)	4 (20)	18 (32.1)	4.14	0.13
**Comorbid ODD, n (%)**	1 (5.6)	3 (16.7)	1 (5)	5 (8.9)	1.95	0.38
**Comorbid learning impairments, n (%)**	1 (5.6)	4 (22.2)	5 (25.0)	10 (17.9)	2.79	0.25
**Mean framewise displacement pre-scrubbing (SD)**	0.089 (0.059)	0.080 (0.042)	0.090 (0.057)	0.087 (0.053)	0.20	0.82
**Mean framewise displacement post-scrubbing (SD)**	0.059 (0.013)	0.053 (0.018)	0.059 (0.022)	0.057 (0.019)	0.53	0.59
**Volumes included after scrubbing, n (SD) - (%) out of 250 acquired volumes)**	224.2 (25.5) - (89.7%)	224.4 (26.2) - (89.7%)	216.0 (34.6) - (86.4%)	221.3 (29.0) - (88.5%)	0.51	0.60
**Participants excluded due to excessive head motion, n (%)**	2 (11.1)	2 (11.1)	3 (15.0)	7 (12.5)	0.19	0.909

Note: Table of relevant phenotypic (sociodemographic, clinical) and imaging quality control characteristics across groups. Results are means (with standard deviations) except for absolute numbers (n), ranges and proportions (%). Statistical differences among groups are given with their corresponding statistical test value (ANOVA F for comparison of means, Pearson's Chi-square for comparison of proportions and Kruskal-Wallis's Chi-square for comparison of medians) and p values. Statistical values are rounded to two decimals. For continuous variables, we conducted descriptive and comparative analyses of means and ranks with both ANOVA and Kruskal-Wallis tests, as the groups are small and heterogeneous. As results were similar, ANOVA are presented for simplicity.

[X] Variables with missing data.

MPH=group 1, on methylphenidate. LDX=group 2, on lisdexamfetamine. X2=Chi-square. PD=professional development/vocational school. ADHD=Attention-deficit/hyperactivity disorder. 15q=Edinburgh Handedness Questionnaire, 15-item version. ADHD-RS.es=ADHD rating scale, Spanish version. CGI-S=Clinical Global Impression, severity subscale. ODD=oppositional-defiant disorder. NA=not applicable. Framewise displacement (FD) refers to the MRI scan at timepoint 1 ("on-medication" for groups 1-3 and "off/pre-medication" for group 4, Naive).

To ascertain whether or not the retained sample was representative of the whole sample of recruited participants, across-group comparisons on these phenotypic and quality control variables including all 68 participants were conducted. Results of these comparisons were similar to those in the analysis of the final retained sample.

Neuroimaging

<u>Across-group comparisons of functional connectivity among brain networks</u>

Across-group comparisons of functional connectivity between pairs of the 10 brain networks extracted through DR based on Smith et al. (4) yielded no statistically significant results after adjustment for multiple statistical comparisons.

<u>Across-group comparisons of functional connectivity among brain nodes</u>

Across-group comparisons of functional connectivity between pairs of the 200 brain nodes extracted based on Schaefer et al. (5) detected 30 pairs of functional connectivity which exceeded the 5% FDR threshold in at least one of the 859 simulations. All of these occurred exclusively in the contrast between LDX and NAIVE groups. Based on the assumption that many of these nominally significant results likely represent false positives, it was decided to focus on the eight pairs that emerged in at least 5% of the simulations. Information about each of the twelve nodes involved in these 8 pairs are shown in Table S2.

Table S2: Salient nodes in the LDX-NAIVE comparisons

Schaefer node number - name	Yeo network designation	MNI coordinates
169 - (Right) lateral prefrontal cortex	Frontoparietal control	46, 24, 26
175 - (Right) lateral dorsal prefrontal cortex	Frontoparietal control	40, 34, 38
180 - (Right) lateral dorsal prefrontal cortex	Frontoparietal control	24, 24, 54
148 - (Right) medial parietal cortex	Salience / Ventral attention	10, -16, 42
39 - (Left) frontal eye field	Dorsal attention	-32 ,-4, 54
133 - (Right) superior parietal lobe	Dorsal attention	32, -74, 32
139 - (Right) posterior cingulate cortex	Dorsal attention	8, -56, 62
75 - (Left) inferior parietal lobe	Default mode	-46, -66, 38
97 - (Left) retrosplenial cortex	Default mode	-10, -56, 12
120 - (Right) node 8	Somatomotor	22, -8, 68
122 - (Right) node 10	Somatomotor	6, -22, 68
112 - (Right) extra-striate superior cortex	Visual	16, -84, 40

Note: Correspondence between Schaefer (5) nodes, Yeo (40) networks, and MNI brain coordinates for the salient nodes involved in statistically reliable comparisons of pairs of networks between LDX (lisdexamfetamine) and NAIVE (treatment-naive) groups.

MNI=Montreal Neurological Institute brain atlas (33) (coordinates x, y, z, in the RAS system).

In the contrast between LDX and NAIVE groups, the average functional connectivity in eight pairs of nodes was significantly lower in the LDX group than in the NAIVE group. Figure S3 displays the 12 brain nodes involved in these eight pairs, which represent 10 distinct brain regions, showing their locations in brain space and the Yeo et al. (40) networks to which each corresponds. The nodes are predominantly located in the right hemisphere and mainly involve the frontoparietal control, attention, and default mode networks, although the somatomotor and visual networks are also represented.

Figure S3: Visual representation of functional connectivity across pairs of brain nodes with significant differences between patients in group LDX vs NAIVE

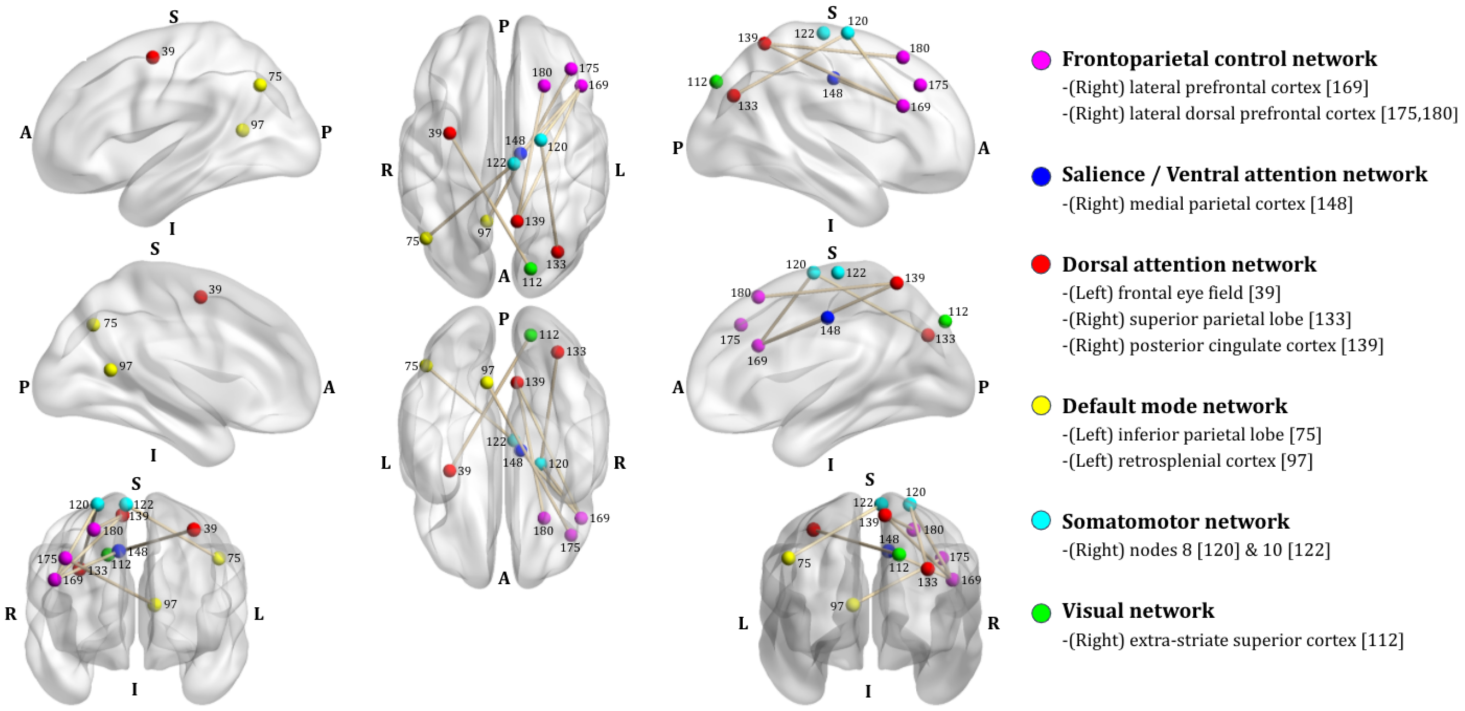

Note: Visual representation of different projections of semi-transparent brains with the hubs and edges of the pairs of brain nodes (numbered as per Schaefer et al. (5) atlas). The correspondence between Schaefer's numbers (in brackets) and node names, as well as between the colors of the edges (nodes) and the brain network they belong to (as per Yeo et al. (40) brain atlas) are indicated on the right section of the figure. A=anterior; P=posterior; L=left; R=right; S=superior; I=inferior. For a correspondence between Schaefer's nodes and brain coordinates (Montreal Neurological Institute, MNI (33)) see Table S2. Graphs generated with BrainNet Viewer software (https://journals.plos.org/plosone/article?id=10.1371/journal.pone.0068910).

Figure S4 shows box plots of the average functional connectivity in the LDX and NAIVE groups for the eight pairs of nodes with prominent between-group differences. As can be clearly appreciated, the LDX group had lower average functional connectivity between those pairs than the NAIVE group; specifically, while the average functional connectivity values in these eight

pairs in the NAIVE group were around zero, they were consistently slightly negative (i.e., showing between-node negative correlations, or anticorrelations) in the LDX group. The specific, highlighted pairs were [(Schaefer et al. (5) numerations), (Yeo et al. (40) network)]:

1. Right frontal eye field (39, dorsal attention) - Right extra-striate superior cortex (112, visual).
2. Left inferior parietal lobe (75, default mode) - Right somatomotor node 10 (122, somatomotor).
3. Right somatomotor node 8 (120, somatomotor) - Right superior parietal lobe (133, dorsal attention).
4. Right somatomotor node 8 (120, somatomotor) - Right lateral prefrontal cortex (169, frontoparietal control).
5. Right posterior cingulate cortex (139, dorsal attention) - Right lateral prefrontal cortex (169, frontoparietal control).
6. Right medial parietal cortex (148, salience / ventral attention) - Right lateral prefrontal cortex (169, frontoparietal control).
7. Left retrosplenial cortex (97, default mode) - Right lateral dorsal prefrontal cortex (175, frontoparietal control).
8. Right posterior cingulate cortex (139, dorsal attention) - Right lateral dorsal prefrontal cortex (180, frontoparietal control).

Three brain nodes were involved in more than one pair:

- Right lateral prefrontal cortex (169, frontoparietal control) was involved in three pairs.
- Right posterior cingulate cortex (139, dorsal attention) was involved in two pairs.
- Right somatomotor node 8 (120, somatomotor) was involved in two pairs.

As categorized according to the Yeo et al. (40) brain networks:

- Nodes in the frontoparietal control network were involved in 5 pairs of significant LDX-NAIVE differences, which included nodes in the dorsal attention (2 pairs), default mode (1 pair), salience / ventral attention (1 pair), and somatomotor (1 pair) networks.
- Nodes in the dorsal attention network were involved in 4 pairs, which included nodes in the frontoparietal control (2 pairs), somatomotor (1 pair), and visual (1 pair).
- Nodes in the default mode network were involved in 2 pairs, which included nodes in the frontoparietal control (1 pair), and the somatomotor (1 pair) networks.
- Nodes in the somatomotor network were involved in 2 pairs, which included nodes in the frontoparietal control (1 pair) and the dorsal attention (1 pair) networks.
- A node in the salience / ventral attention network was involved in 1 pair, which included the frontoparietal control network.
- A node in a visual network was involved in 1 pair, which included the dorsal attention network.

Figure S4: Statistical comparison of functional connectivity across pairs of brain nodes between patients in LDX vs NAIVE groups that were nominally statistically significant in at least 5% of 859 simulations (each with 5000 permutations)

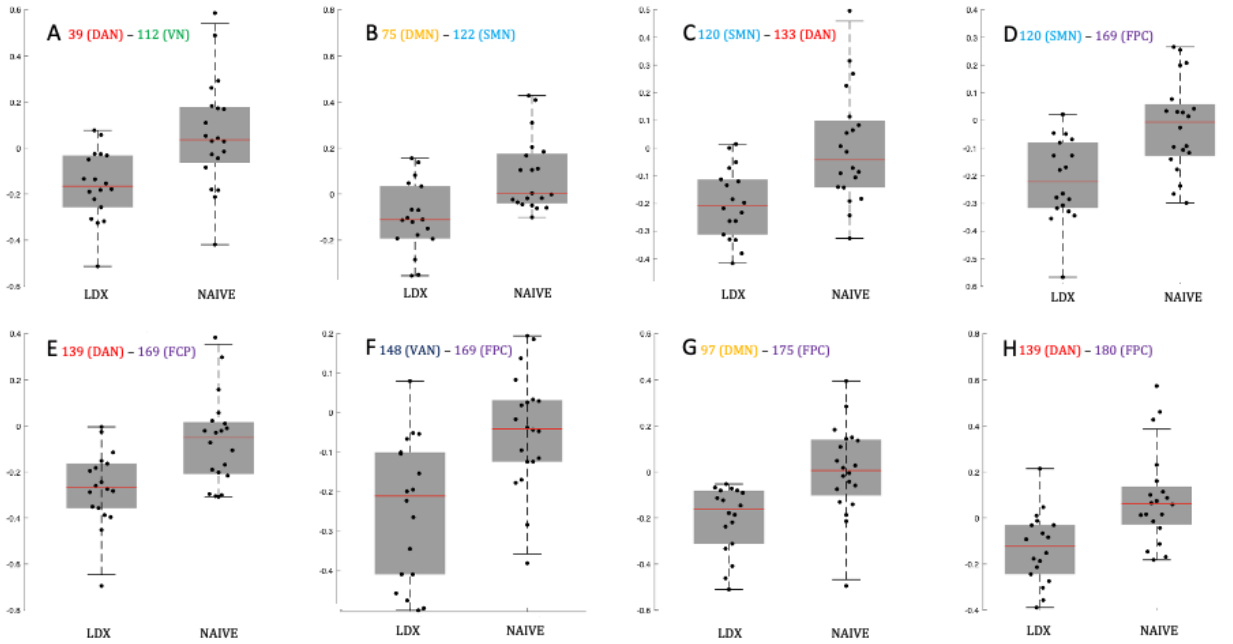

Note: Box plots representing between-group differences in average functional connectivity across pairs of brain nodes (numbered as per the Schaefer et al. (5) atlas; for full correspondence see Table S2 and Figure S3). Only the eight pairs with between-group differences that emerged in at least 5% of simulations were included. Each sub-figure (A-C) presents a comparison between different pairs.

LDX=study group 2, of patients treated with lisdexamfetamine; NAIVE=study group 4, of treatment naive-patients. MPH group (group 1) is not presented here as it did not present statistically-significant differences in comparisons with LDX and NAIVE, although its average functional connectivities (and its representation in box plots) were similar to the connectivities in the NAIVE group.

The corresponding brain networks for each node (as per Yeo et al. (40) brain atlas) is indicated. DAN (red)=Dorsal attention network; VN (green)=Visual network; DMN (yellow)=Default mode network; SMN (light blue)=Somatomotor network; FCP (purple-fuchsia)=Frontoparietal control network; VAN (dark blue)=Salience / Ventral attention network. Graphs generated with MATLAB software.

References

1. Sonuga-Barke EJS, Castellanos FX. Spontaneous attentional fluctuations in impaired states and pathological conditions: a neurobiological hypothesis. *Neurosci Biobehav Rev* (2007) 31:977–986. doi:10.1016/j.neubiorev.2007.02.005
2. Cary RP, Ray S, Grayson DS, Painter J, Carpenter S, Maron L, Sporns O, Stevens AA, Nigg JT, Fair DA. Network Structure among Brain Systems in Adult ADHD is Uniquely Modified by Stimulant Administration. *Cereb Cortex* (2017) 27:3970–3979. doi:10.1093/cercor/bhw209
3. Castellanos FX, Di Martino A, Craddock RC, Mehta AD, Milham MP. Clinical applications of the functional connectome. *NeuroImage* (2013) 80:527–540. doi:10.1016/J.NEUROIMAGE.2013.04.083
4. Smith SM, Fox PT, Miller KL, Glahn DC, Fox PM, Mackay CE, Filippini N, Watkins KE, Toro

R, Laird AR, et al. Correspondence of the brain's functional architecture during activation and rest. *Proc Natl Acad Sci U S A* (2009) 106:13040–13045. doi:10.1073/pnas.0905267106

5. Schaefer A, Kong R, Gordon EM, Laumann TO, Zuo X-N, Holmes AJ, Eickhoff SB, Yeo BTT. Local-Global Parcellation of the Human Cerebral Cortex from Intrinsic Functional Connectivity MRI. *Cereb Cortex N Y N 1991* (2018) 28:3095–3114. doi:10.1093/cercor/bhx179
6. Zalesky A, Fornito A, Bullmore ET. Network-based statistic: Identifying differences in brain networks. *NeuroImage* (2010) 53:1197–1207. doi:10.1016/j.neuroimage.2010.06.041
7. Pereira-Sanchez V, Franco AR, Vieira D, de Castro-Manglano P, Soutullo C, Milham MP, Castellanos FX. Systematic Review: Medication Effects on Brain Intrinsic Functional Connectivity in Patients With Attention-Deficit/Hyperactivity Disorder. *J Am Acad Child Adolesc Psychiatry* (2021) 60:222–235. doi:10.1016/j.jaac.2020.10.013
8. American Psychiatric Association., American Psychiatric Association. DSM-5 Task Force. *Diagnostic and statistical manual of mental disorders : DSM-5.* (2013).
9. Costa ALF, Appenzeller S, Yasuda C-L, Pereira FR, Zanardi VA, Cendes F. Artifacts in brain magnetic resonance imaging due to metallic dental objects. *Med Oral Patol Oral Cirugia Bucal* (2009) 14:E278-82.
10. Poorsattar-Bejeh Mir A, Rahmati-Kamel M. Should the orthodontic brackets always be removed prior to magnetic resonance imaging (MRI)? *J Oral Biol Craniofacial Res* (2016) 6:142–52. doi:10.1016/j.jobcr.2015.08.007
11. Schrantee A, Tamminga HGH, Bouziane C, Bottelier MA, Bron EE, Mutsaerts H-JMM, Zwinderman AH, Groote IR, Rombouts SARB, Lindauer RJL, et al. Age-Dependent Effects of Methylphenidate on the Human Dopaminergic System in Young vs Adult Patients With Attention-Deficit/Hyperactivity Disorder: A Randomized Clinical Trial. *JAMA Psychiatry* (2016) 73:955–962. doi:10.1001/jamapsychiatry.2016.1572
12. An L, Cao X-H, Cao Q-J, Sun L, Yang L, Zou Q-H, Katya R, Zang Y-F, Wang Y-F. Methylphenidate normalizes resting-state brain dysfunction in boys with attention deficit hyperactivity disorder. *Neuropsychopharmacol Off Publ Am Coll Neuropsychopharmacol* (2013) 38:1287–1295. doi:10.1038/npp.2013.27
13. Weiss M, Childress A, Nordbrock E, Adjei A, Kupper R, Mattingly G, Weiss M, Childress A, Nordbrock E, Adjei AL, et al. Characteristics of ADHD Symptom Response/Remission in a Clinical Trial of Methylphenidate Extended Release. *J Clin Med* (2019) 8:461. doi:10.3390/jcm8040461
14. DuPaul GJ, Reid R, Anastopoulos AD, Lambert MC, Watkins MW, Power TJ. Parent and teacher ratings of attention-deficit/hyperactivity disorder symptoms: Factor structure and normative data. *Psychol Assess* (2016) 28:214–225. doi:10.1037/pas0000166
15. Vallejo-Valdivielso M, Soutullo CA, de Castro-Manglano P, Marín-Méndez JJ, Díez-Suárez A. Validación de la versión en español de la escala de evaluación del trastorno por déficit de atención e hiperactividad (ADHD-RS-IV.es) en una muestra española. *Neurología* (2017) doi:10.1016/J.NRL.2017.05.010
16. Busner J, Targum SD. The clinical global impressions scale: applying a research tool in clinical practice. *Psychiatry Edgmont Pa Townsh* (2007) 4:28–37.
17. Dunlop BW, Gray J, Rapaport MH, Dunlop BW, Gray J, Rapaport MH. Transdiagnostic Clinical Global Impression Scoring for Routine Clinical Settings. *Behav Sci* (2017) 7:40.

doi:10.3390/bs7030040
18. Na SD, Burns TG. Wechsler Intelligence Scale for Children-V: Test Review. *Appl Neuropsychol Child* (2016) 5:156–160. doi:10.1080/21622965.2015.1015337
19. Donovick PJ, Burright RG, Burg JS, Gronendyke SJ, Klimczak N, Matthews A, Sardo J. The K-BIT: A screen for IQ in six diverse populations. *J Clin Psychol Med Settings* (1996) 3:131–139. doi:10.1007/BF01996133
20. Carlozzi NE. "Kaufman Brief Intelligence Test," in *Encyclopedia of Clinical Neuropsychology* (New York, NY: Springer New York), 1398–1400. doi:10.1007/978-0-387-79948-3_1062
21. Scarpina F, Tagini S. The Stroop Color and Word Test. *Front Psychol* (2017) 8:557. doi:10.3389/fpsyg.2017.00557
22. Berger I, Slobodin O, Cassuto H. Usefulness and Validity of Continuous Performance Tests in the Diagnosis of Attention-Deficit Hyperactivity Disorder Children. *Arch Clin Neuropsychol* (2016) 32:81–93. doi:10.1093/arclin/acw101
23. Barch DM, Albaugh MD, Avenevoli S, Chang L, Clark DB, Glantz MD, Hudziak JJ, Jernigan TL, Tapert SF, Yurgelun-Todd D, et al. Demographic, physical and mental health assessments in the adolescent brain and cognitive development study: Rationale and description. *Dev Cogn Neurosci* (2018) 32:55–66. doi:10.1016/j.dcn.2017.10.010
24. Oldfield RC. The assessment and analysis of handedness: The Edinburgh inventory. *Neuropsychologia* (1971) 9:97–113. doi:10.1016/0028-3932(71)90067-4
25. Larobina M, Murino L. Medical Image File Formats. *J Digit Imaging* (2014) 27:200–206. doi:10.1007/s10278-013-9657-9
26. Li X, Morgan PS, Ashburner J, Smith J, Rorden C. The first step for neuroimaging data analysis: DICOM to NIfTI conversion. *J Neurosci Methods* (2016) 264:47–56. doi:10.1016/J.JNEUMETH.2016.03.001
27. Gorgolewski KJ, Auer T, Calhoun VD, Craddock RC, Das S, Duff EP, Flandin G, Ghosh SS, Glatard T, Halchenko YO, et al. The brain imaging data structure, a format for organizing and describing outputs of neuroimaging experiments. *Sci Data* (2016) 3:160044. doi:10.1038/sdata.2016.44
28. Cameron C, Sharad S, Brian C, Ranjeet K, Satrajit G, Chaogan Y, Qingyang L, Daniel L, Joshua V, Randal B, et al. Towards Automated Analysis of Connectomes: The Configurable Pipeline for the Analysis of Connectomes (C-PAC). *Front Neuroinformatics* (2013) 7: doi:10.3389/conf.fninf.2013.09.00042
29. Cox RW. AFNI: software for analysis and visualization of functional magnetic resonance neuroimages. *Comput Biomed Res Int J* (1996) 29:162–173. doi:10.1006/cbmr.1996.0014
30. Cox RW. AFNI: What a long strange trip it's been. *NeuroImage* (2012) 62:743–747. doi:10.1016/j.neuroimage.2011.08.056
31. Smith SM, Jenkinson M, Woolrich MW, Beckmann CF, Behrens TEJ, Johansen-Berg H, Bannister PR, De Luca M, Drobnjak I, Flitney DE, et al. Advances in functional and structural MR image analysis and implementation as FSL. *NeuroImage* (2004) 23 Suppl 1:S208-219. doi:10.1016/j.neuroimage.2004.07.051
32. Avants BB, Tustison NJ, Song G, Cook PA, Klein A, Gee JC. A reproducible evaluation of ANTs similarity metric performance in brain image registration. *NeuroImage* (2011) 54:2033–2044. doi:10.1016/j.neuroimage.2010.09.025
33. Mazziotta JC, Toga AW, Evans AC, Fox PT, Lancaster JL. Digital brain atlases. *Trends*

Neurosci (1995) 18:210–211. doi:10.1016/0166-2236(95)93904-C
34. Mandal PK, Mahajan R, Dinov ID. Structural Brain Atlases: Design, Rationale, and Applications in Normal and Pathological Cohorts. *J Alzheimers Dis JAD* (2012) 31:S169. doi:10.3233/JAD-2012-120412
35. Behzadi Y, Restom K, Liau J, Liu TT. A component based noise correction method (CompCor) for BOLD and perfusion based fMRI. *NeuroImage* (2007) 37:90–101. doi:10.1016/j.neuroimage.2007.04.042
36. Friston KJ, Williams S, Howard R, Frackowiak RS, Turner R. Movement-related effects in fMRI time-series. *Magn Reson Med* (1996) 35:346–55.
37. Jenkinson M, Bannister P, Brady M, Smith S. Improved Optimization for the Robust and Accurate Linear Registration and Motion Correction of Brain Images. (2002) doi:10.1006/nimg.2002.1132
38. Pruim RHR, Mennes M, van Rooij D, Llera A, Buitelaar JK, Beckmann CF. ICA-AROMA: A robust ICA-based strategy for removing motion artifacts from fMRI data. *NeuroImage* (2015) 112:267–277. doi:10.1016/j.neuroimage.2015.02.064
39. He H, Liu TT. A geometric view of global signal confounds in resting-state functional MRI. *NeuroImage* (2012) 59:2339–2348. doi:10.1016/j.neuroimage.2011.09.018
40. Yeo BTT, Krienen FM, Sepulcre J, Sabuncu MR, Lashkari D, Hollinshead M, Roffman JL, Smoller JW, Zöllei L, Polimeni JR, et al. The organization of the human cerebral cortex estimated by intrinsic functional connectivity. *J Neurophysiol* (2011) 106:1125–65. doi:10.1152/jn.00338.2011
41. Nichols TE, Das S, Eickhoff SB, Evans AC, Glatard T, Hanke M, Kriegeskorte N, Milham MP, Poldrack RA, Poline J-B, et al. Best practices in data analysis and sharing in neuroimaging using MRI. *Nat Neurosci* (2017) 20:299–303. doi:10.1038/nn.4500
42. Cortese S, Adamo N, Del Giovane C, Mohr-Jensen C, Hayes AJ, Carucci S, Atkinson LZ, Tessari L, Banaschewski T, Coghill D, et al. Comparative efficacy and tolerability of medications for attention-deficit hyperactivity disorder in children, adolescents, and adults: a systematic review and network meta-analysis. *Lancet Psychiatry* (2018) 5:727–738. doi:10.1016/S2215-0366(18)30269-4
